# The effects of ambient temperature exposure on feline fecal metabolome

**DOI:** 10.3389/fvets.2023.1141881

**Published:** 2023-05-26

**Authors:** Olivia Chiu, Moran Tal, Abbinash Sanmugam, Myriam Hesta, Diego E. Gomez, Jeffrey Scott Weese, Adronie Verbrugghe

**Affiliations:** ^1^Department of Clinical Studies, Ontario Veterinary College, University of Guelph, Guelph, ON, Canada; ^2^Department of Morphology, Imaging, Orthopedics, Rehabilitation and Nutrition, Faculty of Veterinary Medicine, Ghent University, Merelbeke, Belgium; ^3^Department of Pathobiology, Ontario Veterinary College, University of Guelph, Guelph, ON, Canada

**Keywords:** fecal metabolites, fecal storage conditions, 1 H nuclear magnetic resonance spectroscopy, amino acids, volatile fatty acids, cat

## Abstract

**Introduction:**

The fecal metabolome provides insight into overall gastrointestinal and microbial health. Methods for fecal sample storage in metabolomics research vary, however, making comparisons within current literature difficult. This study investigated the effect of ambient temperature exposure on microbial-derived metabolites of feline fecal samples.

**Methods:**

Fecal samples were collected from 11 healthy cats from a local boarding facility. Samples were manually homogenized and aliquoted. The first aliquot was frozen at -80°C within 1 hour of defecation, and remaining samples were exposed to ambient temperature for 2, 4, 6, 8, 12, and 24 h prior to freezing at -80°C. Fecal metabolites were quantified using ^1^H NMR spectroscopy. Fifty metabolites were grouped into six categories (27 amino acids, 8 fatty acids, 5 sugars, 3 alcohols, 2 nitrogenous bases, 5 miscellaneous).

**Results:**

Concentrations of 20 out of 50 metabolites significantly differed due to ambient temperature exposure (7 amino acids, 6 fatty acids, 2 alcohols, 1 nitrogenous base, 4 miscellaneous). The earliest detected changes occurred 6 h post-defecation for cadaverine and fumaric acid.

**Discussion:**

This study shows ambient temperature exposure alters the composition of the feline fecal metabolome, but short-term (up to 4 h) exposure prior to storage in the freezer seems to be acceptable.

## 1. Introduction

Fecal matter contains a wide variety of components, including metabolites, which are small molecules reflecting downstream products of the genome, transcriptome, and proteome in the biological system. These fecal metabolites can be used as a sensitive measure of phenotypes when studying environment–gene interactions (1) and reflect nutrient ingestion, digestion, and absorption within the gastrointestinal tract, as well as microbial fermentation within the large intestine (2). Fecal metabolomics, a rapidly expanding area of research, provides a “snapshot” of the entire fecal metabolome (3) and has been applied to assess the microbiota to aid in understanding gastrointestinal physiology and to explore correlations between dysbiosis, fecal metabolic perturbations, and disease pathogenesis (4, 5). Moreover, this emerging technique generates information to discover specific metabolites that could be used as biomarkers for disease (6). Although there is rising popularity in the use of fecal metabolomics, methods of collecting, storing, and analyzing fecal samples have yet to be standardized, particularly in companion animals. In studies collecting fecal samples in field locations, it may not be feasible to collect, process and/or analyze samples immediately. This delay has the potential to alter a sample’s metabolite composition as fecal bacteria metabolize the substrates in the feces (7), leading to equivocal conclusions. This is particularly challenging for studies requiring fecal collection from client-owned companion animals as the samples received are variable due to exposure to different storage temperatures for extended periods of time prior to handling and storage in the laboratory setting. Most studies investigating the effect of room temperature exposure on the metabolomic profile have been completed in humans. Gratton et al. (8) observed alterations in fecal metabolite concentrations mainly within 1–5 h of room temperature exposure in adult humans, while Liang et al. (9) observed minimal effect on the fecal metabolome following 4 h of room temperature exposure in 34-month-old children. The time course of changes to fecal metabolite concentrations during room temperature exposure in companion animals is currently unclear. This research would help to standardize sample collection and storage methods for future research aiming to investigate the role of fecal metabolites in disease and dysbiosis in companion animals. The present study aimed to determine the effect of room temperature exposure across a 24 h period on metabolite concentrations in feline feces using a targeted metabolomics approach. We hypothesized that significant changes in the fecal metabolomic profile would occur mainly within the first 6 h of exposure to room temperature, similar to previous results in humans (8).

## 2. Methods and materials

### 2.1. Animals

Eleven healthy adult cats housed at a boarding facility in Guelph, Ontario were included in this study. The cats were deemed healthy based on information provided by the cat owners prior to boarding. All cats were fed traditional commercial diets, however, the specific details of their diets were not recorded. In consultation with the University of Guelph Animal Care and Use Committee, an Animal Use Protocol was deemed unnecessary for a study performing no animal-related procedures other than collecting fecal samples from cat litter boxes. Litter was replaced once a day, in the morning before feeding.

### 2.2. Sample collection

Fecal samples were collected as part of a previously published fecal microbiota study (10). Cat at the boarding facility were observed by facility personnel daily. Fecal samples were collected from each cat within 15 min of defecation. Samples were then taken immediately to the laboratory unrefrigerated, then they were manually homogenized and aliquoted into seven different 200 mg samples. Manual homogenization involved kneading the feces for homogenized textures and layers that may contain different microbial composition, structure, and metabolites. One aliquot was frozen at −80°C within 1 h after defecation. All other aliquots were kept at room temperature (20 to 23°C) in a biosafety cabinet^1^ before freezing at −80°C at the specified time points: 2, 4, 6, 8, 12, and 24 h after sample collection. All samples were kept frozen at −80°C until further analysis was completed. The time points designated for this study were chosen based on data from a previous study completed with humans, suggesting that metabolome changes mainly begin to occur in the first 12 h after sample collection (8).

### 2.3. Nuclear magnetic resonance analysis

The tubes containing the manually homogenized samples were sent frozen on dry ice to The Metabolomic Innovation Centre^2^ for quantitative NMR spectroscopy (4, 8, 11–13). Samples were thawed, then 600 μL of high performance liquid chromatography water was added to each sample, and they were vortexed, shaken, centrifuged, and filtered. A 200 μL aliquot of each sample was then placed in a 1.5 mL Eppendorf tube. Fifty μL of a standard buffer solution was then added (54% D2O: 46% 1.75 mM KH_2_PO_4_ (phosphate buffered saline) pH 7.0 v/v containing 5.84 mM DSS (2,2-dimethyl-2-silcepentane-5-sulphonate), and 0.1% NaN_3_ in H_2_O). The total sample, now containing 250 μL, was then transferred for spectral analysis using the 3 mm SampleJet NMR tube. The ^1^H-NMR spectrum was then collected on the 700 MHz Avance (Bruker) spectrometer, which was equipped with a 5 mm HCN Z-gradient pulsed-field gradient cryoprobe. The first transient of NOESY pre-saturation pulse sequence (noesy1dpr) was used to acquire the ^1^H-NMR spectra at 25°C and was chosen due to the high degree of quantitative accuracy (14). Free induction decays were all zero-filled to 250 K data points, and the singlet produced from the DSS methyl groups was used as an internal standard for chemical shift referencing. This referencing was set to zero parts per million for quantification of all ^1^H-NMR spectra. This was processed and analyzed using the Chenomx NMR Suite Professional software package version 8.4 (Chemomx Inc., Edmonton, AB). Two spectroscopists then inspected each spectrum to minimize misidentification and misquantification of compounds. All visible peaks were annotated with a compound name based on the corresponding proton resonance (14). The metabolites analyzed were categorized into six metabolite groups based on descriptions of each compound available via MetaboAnalyst^3^ pathway analysis and the National Library of Medicine PubChem^4^ platforms. The six groups were: (1) amino acids, amines, and their metabolites; (2) fatty acids; (3) sugars and sugar metabolites; (4) alcohols; (5) nitrogenous bases; and (6) other metabolites. The metabolite group classifications and compounds analyzed are described in Table 1.

**Table 1:** Metabolite group classifications and compounds that were analyzed in feline fecal samples (*n* = 11) using nuclear magnetic resonance spectrometry.

Metabolite group	Compounds
Amino acids, amines, and their metabolites	Asparagine, creatine, glutamine, methylamine, 4-aminobutyrate, alanine, aspartate, betaine, dimethylamine, glycine, glutamic acid, histidine, isoleucine, leucine, lysine, methionine, putrescine, taurine, threonine, tryptophan, tyrosine, valine, cadaverine, creatinine, phenylacetate, phenylalanine, trimethylamine
Fatty acids	Acetic acid, propionate, 3-hydroxyisobutyrate, butyrate, 3-hydroxyisovaleric acid, isobutyric acid, isovaleric acid, valerate
Sugars and sugar metabolites	D-glucose, D-galactose, L-fucose, succinate, L-lactic acid
Alcohols	Ethanol, methanol, glycerol
Nitrogenous bases	Uracil, xanthine
Other metabolites	Choline, acetoin, acetone, formate, fumaric acid

### 2.4. Statistical analysis

Statistical analyses were completed using Statistical Analysis System (SAS) software (SAS Institute Inc.). If data for metabolite concentrations were not normally distributed (determined via a Shapiro–Wilk test), a logarithmic transformation was performed before further analysis. For metabolites with normally distributed data and for transformed data, repeated measures analysis of variance (ANOVA) was used to detect differences across time for each metabolite, using a 5% significance level (*p* < 0.05). Following repeated measures ANOVA, post-hoc paired t-tests with Bonferroni corrections were used to detect differences between each specific time point. A Bonferroni correction was used because when multiple hypotheses are tested (e.g., whether concentrations differed between each time point in the present study), the probability of a type I error increases (15). The Bonferroni correction compensates for this potential of a type I error by testing each individual hypothesis at an adjusted significance level of α divided by the total number of hypotheses (15). In pairwise comparisons, SAS software applies the Bonferroni correction by adjusting the raw *p*-values such that the statistical output can be read using a 5% significance level (*p* < 0.05). If the values were still not normally distributed after logarithmic transformation, a Friedman’s test (for non-parametric analyses) was performed on the non-transformed data using a 5% significance level (*p* < 0.05) to detect differences across time. Wilcoxon tests with Bonferroni corrections were then performed on these non-transformed data to compare metabolite concentrations between each specific time point. Normally distributed, untransformed data are reported as the mean ± standard deviation. Transformed data are reported as the back-transformed mean, and back-transformed lower and upper limits. Non-parametrically analyzed data are reported as the median, and minimum and maximum values. The heatmap was constructed using conditional formatting in Microsoft Excel.

## 3. Results

Fifty metabolites were quantified: 27 amino acids and amines, eight fatty acids, five sugars and sugar metabolites, three alcohols, two nitrogenous bases, and five other metabolites (Table 1).

**Figure 1:**
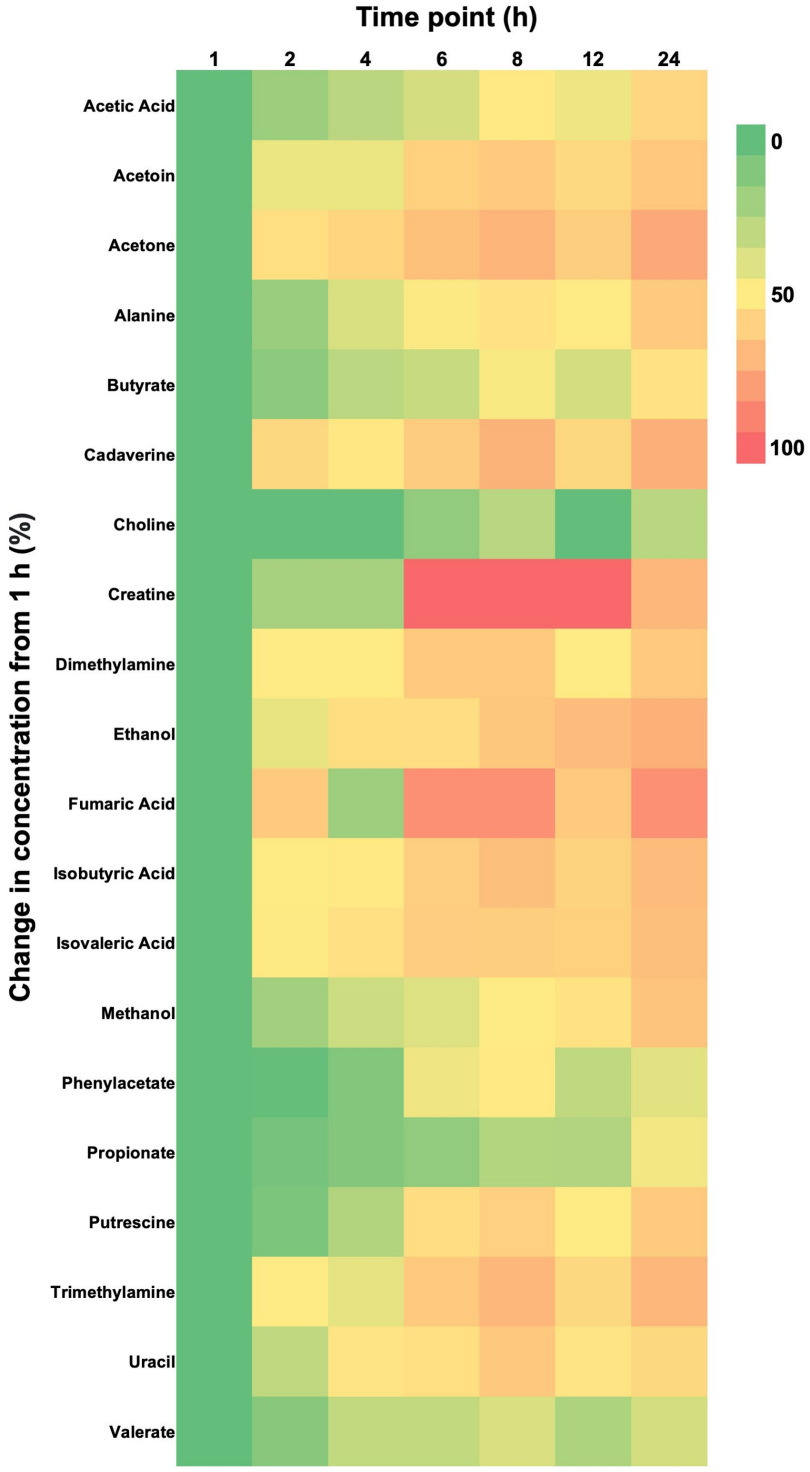
Heat map showing percent changes from baseline (1 h) of fecal metabolite concentrations in feline fecal samples (*n* = 11) for metabolites that experienced a significant effect of time over 24 h of storage at room temperature as shown by repeated measures ANOVA or Friedman’s test. Metabolites are arranged in alphabetical order.

Repeated measures ANOVA (normally distributed and log-transformed data) and Friedman’s tests (non-normally distributed data) showed 20 of 50 (40%) metabolites significantly differed in concentration over time when exposed to room temperature (Tables 2–4; Figure 1). These included seven amino acids and amines, six fatty acids, two alcohols, one nitrogenous base, and four other metabolites. Specifically, repeated measures ANOVA and Friedman’s tests showed alanine, dimethylamine, putrescine, cadaverine, phenylacetate, trimethylamine, acetic acid, propionate, butyrate, isobutyric acid, isovaleric acid, valerate, ethanol, methanol, uracil, acetoin, acetone, and choline all increased in concentration over time. Fecal creatine and fumaric acid concentrations decreased over time.

**Table 2:** The fecal concentrations of metabolites with untransformed data in feline fecal samples (*n* = 11) across time points over 24 h of storage at room temperature.

Compound	Time post-collection exposed to room temperature							*p*-value
	1 h	2 h	4 h	6 h	8 h	12 h	24 h	
Acetic acid	52.78 ± 12.68^a^	59.94 ± 18.89^a^	64.59 ± 15.49^ab^	69.16 ± 17.92^ab^	79.98 ± 17.81^bc^	74.58 ± 15.52^ab^	94.42 ± 20.22^b^	<0.001
Propionate	26.91 ± 8.41^ab^	27.97 ± 10.67^a^	28.91 ± 8.17^ab^	29.90 ± 12.38^a^	32.47 ± 11.70^ab^	32.10 ± 12.58^ab^	38.51 ± 15.23^b^	<0.001
Creatine	0.08 ± 0.03^a^	0.07 ± 0.03^a^	0.07 ± 0.04^a^	0.04 ± 0.02^a^	0.04 ± 0.02^a^	0.04 ± 0.03^a^	0.05 ± 0.03^a^	0.009
Choline	0.09 ± 0.03^a^	0.09 ± 0.04^a^	0.09 ± 0.04^a^	0.10 ± 0.05^a^	0.11 ± 0.04^a^	0.09 ± 0.05^a^	0.11 ± 0.04^a^	0.045
Methylamine	0.33 ± 0.16^a^	0.35 ± 0.16^a^	0.35 ± 0.18^a^	0.37 ± 0.17^a^	0.39 ± 0.17^a^	0.36 ± 0.18^a^	0.39 ± 0.16^a^	0.126
Glutamine	0.89 ± 0.50^a^	0.80 ± 0.35^a^	0.84 ± 0.48^a^	0.95 ± 0.45^a^	1.07 ± 0.46^a^	0.98 ± 0.50^a^	0.93 ± 0.63^a^	0.127
Asparagine	0.85 ± 0.41^a^	0.73 ± 0.39^a^	0.83 ± 0.32^a^	0.80 ± 0.37^a^	0.77 ± 0.28^a^	0.76 ± 0.36^a^	0.67 ± 0.25^a^	0.340
D-glucose	2.82 ± 1.41^a^	3.28 ± 2.16^a^	3.12 ± 2.13^a^	3.04 ± 1.80^a^	3.21 ± 1.74^a^	2.78 ± 1.36^a^	2.58 ± 1.58^a^	0.679

*p*-values displayed in the rightmost column are from repeated measures ANOVAs. Letters denote significant difference between time points (*p* < 0.05), where any shared letters show no differences between timepoints and having no shared letters show differences between timepoints. Values are reported as the mean ± standard deviation.

**Table 3:** The fecal concentrations of metabolites with log-transformed data in feline fecal samples (*n* = 11) across time points over 24 h of storage at room temperature.

Compound	Time post-collection exposed to room temperature							*p*-value
	1 h	2 h	4 h	6 h	8 h	12 h	24 h	
Putrescine	0.55 (0.35–0.86)^ab^	0.58 (0.41–0.83)^a^	0.66 (0.40–1.09)^abc^	0.92 (0.57–1.48)^abc^	1.04 (0.73–1.48)^c^	0.82 (0.55–1.22)^abc^	1.10 (0.75–1.61)^bc^	<0.001
Butyrate	18.57 (13.99–24.65)^a^	20.41 (17.05–24.43)^ab^	22.82 (18.62–27.98)^abc^	23.49 (18.00–30.64)^abc^	27.10 (21.74–33.77)^ac^	24.28 (18.38–32.06)^abc^	29.61 (22.69–38.65)^c^	<0.001
Acetoin	0.15 (0.10–0.23)^a^	0.21 (0.15–0.29)^ab^	0.21 (0.15–0.30)^abc^	0.28 (0.22–0.37)^abc^	0.30 (0.23–0.38)^abc^	0.26 (0.21–0.33)^bc^	0.31 (0.24–0.40)^c^	<0.001
Methanol	0.32 (0.23–0.44)^a^	0.37 (0.25–0.57)^ab^	0.41 (0.27–0.61)^ab^	0.43 (0.28–0.66)^abc^	0.48 (0.31–0.76)^bc^	0.51 (0.34–0.77)^bc^	0.68 (0.38–1.20)^c^	0.001
Dimethylamine	0.02 (0.02, 0.03)^a^	0.03 (0.02, 0.04)^a^	0.03 (0.02, 0.04)^a^	0.04 (0.02–0.05)^a^	0.04 (0.03, 0.05)^a^	0.03 (0.02, 0.05)^a^	0.04 (0.03, 0.06)^a^	0.003
Alanine	2.28 (1.51, 3.43)^a^	2.58 (1.71, 3.89)^a^	3.03 (2.04, 4.51)^a^	3.35 (1.89, 5.94)^a^	3.64 (2.12, 6.25)^a^	3.43 (2.37, 4.96)^a^	4.58 (3.00, 6.97)^a^	0.004
Ethanol	0.67 (0.34–1.29)^ab^	0.93 (0.55–1.57)^a^	1.11 (0.68–1.82)^abc^	1.12 (0.53–2.35)^abd^	1.38 (0.72–2.62)^bcd^	1.56 (0.83–2.92)^ce^	1.81 (0.86–3.80)^de^	0.006
4-Aminobutyrate	0.13 (0.10–0.16)^a^	0.20 (0.12–0.32)^a^	0.23 (0.13–0.39)^a^	0.25 (0.13–0.500)^a^	0.28 (0.16–0.51)^a^	0.21 (0.09–0.49)^a^	0.26 (0.11–0.60)^a^	0.068
Valine	1.13 (0.61–2.09)^a^	1.36 (0.83–2.21)^a^	1.73 (1.14–2.63)^a^	1.88 (0.99–3.58)^a^	2.12 (1.23–3.63)^a^	1.76 (1.12–2.78)^a^	2.26 (1.38–3.71)^a^	0.122
3-Hydroxy-isobutyrate	0.55 (0.39–0.76)^a^	0.62 (0.42–0.92)^a^	0.84 (0.60–1.18)^a^	0.80 (0.52–1.22)^a^	0.60 (0.36–0.99)^a^	0.66 (0.42–1.03)^a^	0.83 (0.54–1.27)^a^	0.161
Tyrosine	0.69 (0.42–1.13)^a^	0.77 (0.49–1.20)^a^	0.87 (0.55–1.38)^a^	1.03 (0.61–1.73)^a^	1.03 (0.62–1.70)^a^	0.91 (0.61–1.36)^a^	0.92 (0.55–1.53)^a^	0.192
Succinate	0.08 (0.04–0.16)^a^	0.06 (0.04–0.10)^a^	0.06 (0.03–0.10)^a^	0.05 (0.02–0.12)^a^	0.07 (0.04–0.12)^a^	0.06 (0.03–0.14)^a^	0.11 (0.04–0.28)^a^	0.220
Histidine	0.22 (0.15–0.34)^a^	0.25 (0.15–0.41)^a^	0.24 (0.16–0.36)^a^	0.30 (0.20–0.46)^a^	0.27 (0.16–0.45)^a^	0.26 (0.16–0.45)^a^	0.21 (0.14–0.32)^a^	0.247
Threonine	0.84 (0.56–1.26)^a^	0.91 (0.69–1.21)^a^	0.95 (0.72–1.25)^a^	1.01 (0.67–1.51)^a^	0.97 (0.72–1.30)^a^	0.84 (0.63–1.11)^a^	0.73 (0.5–1.07)^a^	0.260
Tryptophan	0.21 (0.15–0.28)^a^	0.22 (0.16–0.29)^a^	0.22 (0.16–0.31)^a^	0.28 (0.21–0.37)^a^	0.25 (0.18–0.35)^a^	0.22 (0.17–0.30)^a^	0.25 (0.16–0.39)^a^	0.270
L-fucose	0.42 (0.29–0.61)^a^	0.39 (0.27–0.57)^a^	0.27 (0.17–0.42)^a^	0.33 (0.22–0.52)^a^	0.35 (0.23–0.52)^a^	0.28 (0.22–0.35)^a^	0.27 (0.16–0.46)^a^	0.270
Methionine	0.50 (0.29–0.86)^a^	0.56 (0.35–0.89)^a^	0.61 (0.39–0.94)^a^	0.73 (0.45–1.18)^a^	0.64 (0.38–1.07)^a^	0.64 (0.42–0.96)^a^	0.73 (0.44–1.21)^a^	0.281
Leucine	1.32 (0.82–2.12)^a^	1.47 (0.90–2.41)^a^	1.69 (1.07–2.67)^a^	1.79 (0.96–3.34)^a^	1.82 (1.07–3.10)^a^	1.63 (1.03–2.58)^a^	1.85 (1.12–3.03)^a^	0.345
Glycine	1.26 (0.88–1.80)^a^	1.19 (0.83–1.73)^a^	1.32 (0.92–1.87)^a^	1.43 (0.83–2.48)^a^	1.46 (0.89–2.39)^a^	1.46 (1.00–2.14)^a^	1.44 (0.84–2.48)^a^	0.415
D-galactose	0.53 (0.37–0.77)^a^	0.36 (0.19–0.69)^a^	0.44 (0.31–0.64)^a^	0.55 (0.39–0.78)^a^	0.45 (0.30–0.69)^a^	0.45 (0.35–0.59)^a^	0.38 (0.22–0.65)^a^	0.415
Lysine	1.51 (0.77–3.00)^a^	1.80 (1.14–2.84)^a^	1.97 (1.22–3.18)^a^	1.84 (0.99–3.42)^a^	2.03 (0.91–4.52)^a^	1.86 (0.83–2.89)^a^	1.81 (0.82–3.99)^a^	0.438
Aspartate	1.26 (0.83–1.91)^a^	1.38 (1.03–1.85)^a^	1.47 (1.01–2.12)^a^	1.67 (1.09–2.56)^a^	1.51 (0.98–2.33)^a^	1.48 (1.09–1.99)^a^	1.37 (0.89–2.11)^a^	0.524
Isoleucine	0.47 (0.25–1.12)^a^	0.56 (0.29–1.08)^a^	0.44 (0.17–1.15)^a^	0.70 (0.33–1.48)^a^	0.50 (0.25–1.01)^a^	0.55 (0.25–1.23)^a^	0.65 (0.30–1.38)^a^	0.539
Betaine	0.02 (0.02–0.03)^a^	0.03 (0.02–0.04)^a^	0.03 (0.02–0.04)^a^	0.02 (0.02–0.03)^a^	0.04 (0.02–0.06)^a^	0.03 (0.02–0.04)^a^	0.03 (0.02–0.05)^a^	0.587
Glutamic acid	2.43 (1.45–4.07)^a^	2.38 (1.62–3.48)^a^	2.60 (1.70–3.99)^a^	2.76 (1.62–4.71)^a^	2.79 (1.67–4.64)^a^	2.50 (1.76–3.55)^a^	2.57 (1.40–4.71)^a^	0.635
Taurine	0.35 (0.10–1.18)^a^	0.39 (0.13–1.12)^a^	0.41 (0.14–1.20)^a^	0.39 (0.12–1.77)^a^	0.44 (0.14–1.41)^a^	0.30 (0.08–1.06)^a^	0.37 (0.10–1.35)^a^	0.901

*p*-values displayed in the rightmost column are from repeated measures ANOVAs. Letters denote significant difference between time points (*p* < 0.05), where any shared letters show no differences between timepoints and having no shared letters show differences between timepoints. Values are reported as the back-transformed mean, and back-transformed lower and upper limits.

**Table 4:** The fecal concentrations of metabolites with non-parametrically analyzed data in feline fecal samples (*n* = 11) across time points over 24 h of storage at room temperature.

Compound	Time post-collection exposed to room temperature							*p*-value
	1 h	2 h	4 h	6 h	8 h	12 h	24 h	
Phenylacetate	1.49 (0.12–5.05)^a^	1.50 (0.16–3.71)^a^	1.60 (0.19–4.54)^ab^	2.11 (0.23–3.43)^ab^	2.26 (0.26–3.92)^b^	1.85 (0.20–3.81)^ab^	2.03 (0.21–3.91)^b^	<0.001
Isobutyric acid	1.44 (0.11–7.25)^a^	2.12 (0.32–4.76)^ab^	2.18 (0.33–6.28)^abc^	2.74 (0.41–6.20)^abc^	3.24 (0.17–6.43)^bc^	2.64 (0.23–7.77)^bc^	3.35 (0.35–8.37)^c^	<0.001
Isovaleric acid	2.36 (0.25–11.09)^a^	3.49 (0.28–7.51)^ab^	3.84 (0.31–10.26)^abc^	4.58 (0.21–10.06)^abc^	4.55 (0.30–10.00)^bc^	4.40 (0.35–12.47)^bc^	5.27 (0.32–12.28)^c^	<0.001
Fumaric acid	0.09 (0.05–0.19)^a^	0.06 (0.03–0.15)^ab^	0.08 (0.04–0.10)^abc^	0.05 (0.02–0.15)^bc^	0.05 (0.03–0.13)^bc^	0.06 (0.02–0.12)^bc^	0.05 (0.02–0.10)^c^	<0.001
Acetone	0.14 (0.06–0.29)^a^	0.23 (0.07–0.45)^a^	0.25 (0.07–0.51)^ab^	0.31 (0.07–0.44)^ab^	0.36 (0.04–0.70)^b^	0.27 (0.07–0.46)^ab^	0.43 (0.08–0.53)^b^	0.001
Cadaverine	0.64 (0.25–3.77)^a^	1.13 (0.62–2.26)^ab^	0.98 (0.44–3.61)^ab^	1.25 (0.76–4.44)^b^	1.69 (0.94–5.07)^b^	1.13 (0.17–3.88)^ab^	1.79 (0.25–2.75)^b^	0.001
Trimethylamine	0.08 (0.01–0.17)^a^	0.12 (0.02–0.19)^ab^	0.11 (0.03–0.23)^ab^	0.16 (0.02–0.19)^ab^	0.20 (0.02–0.33)^b^	0.14 (0.02–0.26)^ab^	0.20 (0.01–0.30)^b^	0.003
Valerate	6.67 (0.61–10.75)^ab^	7.24 (0.53–9.68)^a^	8.34 (0.69–10.52)^ab^	8.33 (0.59–11.28)^ab^	8.89 (0.91–11.76)^ab^	7.92 (0.83–10.41)^ab^	8.72 (1.56–12.02)^b^	0.007
Uracil	0.33 (0.14–1.25)^a^	0.41 (0.16–1.39)^a^	0.52 (0.28–1.46)^a^	0.54 (0.09–1.13)^a^	0.67 (0.21–1.84)^a^	0.52 (0.14–1.36)^a^	0.58 (0.19–1.80)^a^	0.011
Glycerol	2.59 (1.37–3.95)^a^	2.86 (1.87–4.19)^a^	2.86 (1.68–3.93)^a^	3.07 (1.94–3.52)^a^	3.01 (1.39–4.24)^a^	2.69 (1.39–3.52)^a^	2.38 (1.32–2.98)^a^	0.082
Formate	0.19 (0.13–2.14)^a^	0.16 (0.12–2.12)^a^	0.16 (0.05–0.10)^a^	0.19 (0.13–2.10)^a^	0.18 (0.12–2.47)^a^	0.36 (0.13–1.98)^a^	0.16 (0.13–2.06)^a^	0.131
L-lactic acid	0.26 (0.09–54.01)^a^	0.21 (0.10–57.96)^a^	0.26 (0.11–33.25)^a^	0.22 (0.11–67.31)^a^	0.33 (0.18–67.34)^a^	0.24 (0.09–61.55)^a^	0.38 (0.15–68.84)^a^	0.163
Phenylalanine	0.63 (0.30–2.45)^a^	0.67 (0.33–2.65)^a^	0.73 (0.52–3.25)^a^	0.92 (0.37–3.13)^a^	0.93 (0.43–3.42)^a^	0.78 (0.51–3.26)^a^	0.81 (0.38–5.04)^a^	0.254
Xanthine	0.03 (0.02–0.10)^a^	0.04 (0.02–0.06)^a^	0.04 (0.03–0.17)^a^	0.04 (0.03–0.06)^a^	0.04 (0.02–0.06)^a^	0.03 (0.02–0.07)^a^	0.04 (0.02–0.08)^a^	0.357
Creatinine	0.21 (0.16–0.73)^a^	0.22 (0.17–0.80)^a^	0.23 (0.19–0.72)^a^	0.22 (0.14–0.62)^a^	0.24 (0.17–0.48)^a^	0.24 (0.16–0.65)^a^	0.21 (0.16–0.54)^a^	0.882
3-Hydroxy-isovaleric acid	0.03 (0.01–0.04)^a^	0.03 (0.01–0.04)^a^	0.03 (0.00–0.05)^a^	0.03 (0.00–0.04)^a^	0.03 (0.01–0.05)^a^	0.02 (0.01–0.06)^a^	0.02 (0.01–0.10)^a^	0.961

*p*-values displayed in the rightmost column are from Friedman’s tests. Letters denote significant difference between time points (*p* < 0.05), where any shared letters show no differences between timepoints and having no shared letters show differences between timepoints. Values are reported as the median, minimum and maximum.

**Figure 2:**
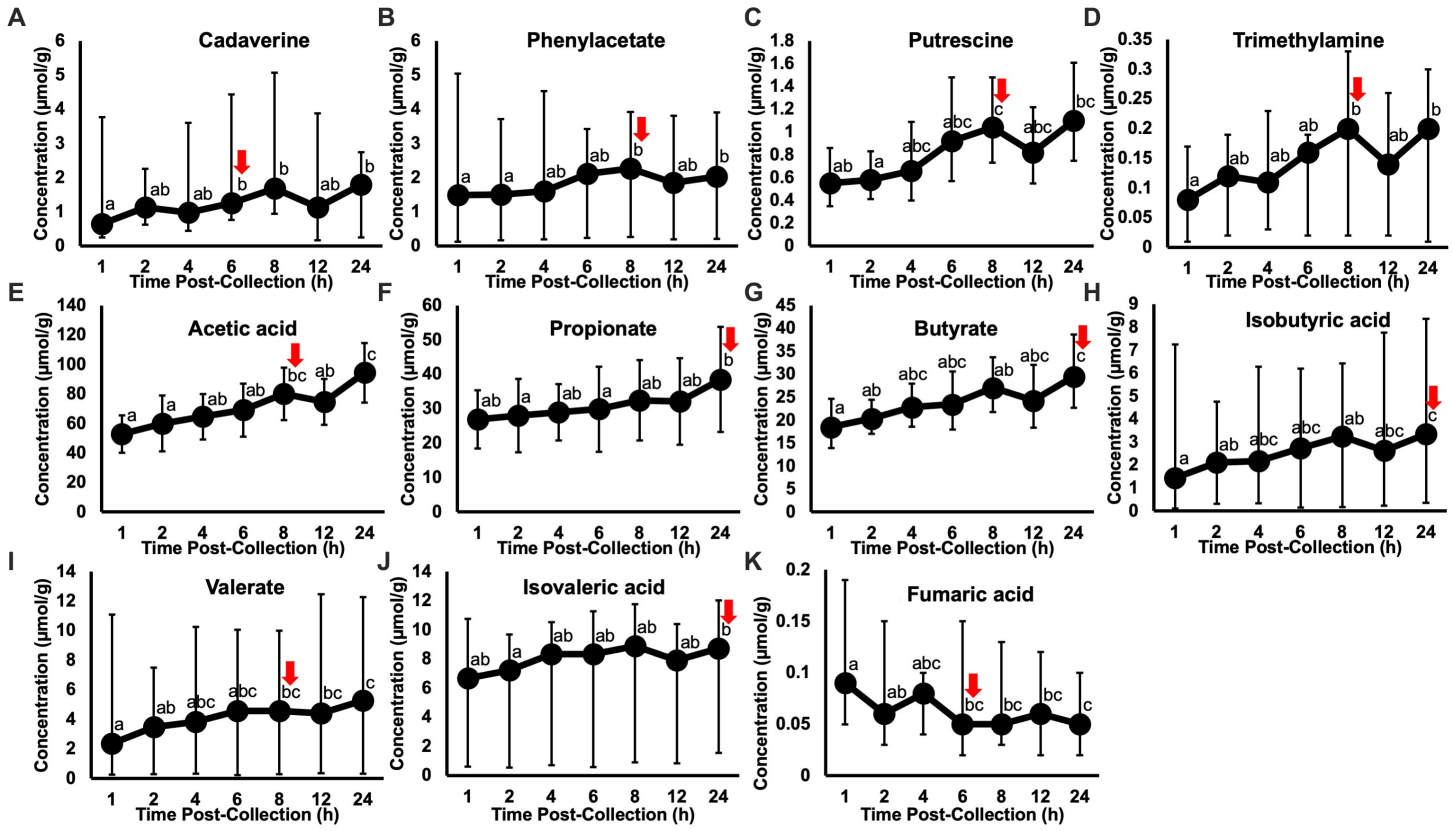
Metabolite concentrations classified as amino acids, amines and their metabolites **(A–D)** and fatty acids **(E–J)**, and fumaric acid (an “other metabolite” involved indirectly in sugar metabolism) that significantly changed within 24 h of room temperature exposure after fecal sample collection in healthy adult cats (*n* = 11). Letters denote a significant difference between time points (*p* < 0.05), where shared letters show no difference between timepoints and no shared letters show a difference between timepoints. Red arrows indicate the time point at which the first change in concentration occurred. The earliest changes in metabolite concentrations were detected at 6 h, in cadaverine **(A)** and fumaric acid **(K)**. Putrescine and butyrate are displayed as the back-transformed mean, with error bars representing the back-transformed upper and lower limits (log-transformed data). Cadaverine, phenylacetate, trimethylamine, isobutyric acid, isovaleric acid, valerate, and fumaric acid are displayed as the median, with error bars representing the maximum and minimum (non-parametrically analyzed data). Acetic acid and propionate are displayed as the mean ± standard deviation (normally distributed data).

Post-hoc analyses (paired t-tests for normally distributed and log-transformed data, and Wilcoxon tests for non-normally distributed data) showed significant differences between individual time points in 15 metabolites: cadaverine (Figure 2A), phenylacetate (Figure 2B), putrescine (Figure 2C), trimethylamine (Figure 2D), acetic acid (Figure 2E), propionate (Figure 2F), butyrate (Figure 2G), isobutyric acid (Figure 2H), valerate (Figure 2I), isovaleric acid (Figure 2J), fumaric acid (Figure 2K) ethanol, methanol, acetoin, and acetone (also see heatmap in Figure 1). The time course of changes in metabolites classified as amino acids, amines, and their metabolites and fatty acids that significantly differed between time points, and fumaric acid (an “other metabolite” with a role in sugar metabolism) are presented in Figure 2. The earliest detected changes occurred at 6 h after room temperature exposure with an increase in cadaverine and a decrease in fumaric acid. At 8 h post-collection, phenylacetate, trimethylamine, putrescine, acetic acid, isobutyric acid, isovaleric acid, ethanol, methanol, and acetone began increasing. Acetoin started increasing at 12 h post-collection. Propionate, butyrate, and valerate concentrations increased at 24 h post-collection.

## 4. Discussion

The fecal metabolome provides insight into an organism’s gastrointestinal and overall health. Exposure of fecal samples to room temperature has the potential to alter fecal metabolite composition as substrates are metabolized by fecal bacteria (7), skewing the results of metabolomic analyses. These potential changes in fecal metabolites become an issue in field locations and when fecal samples are collected from client-owned companion animals, in which the duration of exposure to room temperature may vary. Therefore, standardization of the methods for collection and storage of fecal samples from companion animals is needed, specifically regarding room temperature exposure. The purpose of the present study was to determine the effect of room temperature exposure across a 24 h period on metabolite concentrations in feline fecal samples. We hypothesized that significant changes in the fecal metabolomic profile would occur within the first 6 h of exposure to room temperature, similar to previous results in humans (8).

Our hypothesis was partly correct. Concentrations of more than half of the fecal metabolites remained stable with exposure to room temperature across the time points investigated within 24 h post-collection, but some metabolites did change over time. Notably, fecal concentrations of cadaverine (an amine metabolite) and fumaric acid (a compound involved in sugar metabolism through the citric acid cycle) increased and decreased, respectively, at 6 h post-collection. Other metabolites in the amino acid/amine group (i.e., putrescine, phenylacetate, and trimethylamine), and some in the fatty acid group (i.e., acetic acid, isobutyric acid, and isovaleric acid) increased 8 h post-collection. Three other metabolites in the fatty acid group (i.e., propionate, butyrate, and valerate) increased at 24 h post-collection. These findings are like those of Gratton et al. (8) in humans, in which changes in fecal metabolite concentrations occurred 1 to 5 h into exposure to room temperature, particularly in amino acids and volatile fatty acids. Another study on 34-month-old children observed minimal effect on fecal metabolite concentrations within 4 h of exposure to room temperature (9), similar to the results of the present study. Both previous studies determined that microbial fermentation was the main source of these time-related changes in metabolites.

The present study’s findings suggest that in feline feces, amino acid and amine metabolites generally change with room temperature exposure earlier than volatile fatty acids. This is likely a result of bacterial fermentation in the feces, with amino acid and amine metabolites being products of protein fermentation, and volatile fatty acids being products of carbohydrate fermentation. Although diet history was unknown for the cats enrolled in the present study, cats are historically obligate carnivores, consuming protein-rich diets. The diversity of microorganisms found in the feline gastrointestinal tract is thus regulated by carbon sources derived largely from protein fermentation (16), with intestinal bacteria selecting for the associated substrates (7). Amino acids are more readily available to the bacterial population present, and subsequently metabolize with exposure to oxygen at room temperature due to continued bacterial activity, producing amino acid metabolites. The fecal metabolites present depend on the available substrates and bacteria in the gut, which are affected by diet. If a host consumes more carbohydrates compared to protein, less protein will be available in the gut and carbohydrate fermentation will dominate. Conversely, if carbohydrate consumption is depleted, a shift to protein fermentation will occur. This coincides with shifts in relative abundances of microbial species, which can alter the gut microbiota and metabolome (17). Higher alpha diversity (a measure of species richness and evenness within a microbial community, reflecting the variety and distribution of species in a given area) was found in cats consuming a higher protein diet compared to a lower protein (and thus higher carbohydrate) diet (18). Fecal metagenomics also revealed upregulation of amino acid and urea metabolism, and mucin foraging pathways, and a positive association between amino acid catabolism and mucin degradation with a high protein diet. Moreover, cats fed the higher protein diet presented higher fecal concentrations of branched chain fatty acids and ammonia and a higher fecal pH, suggesting more proteolytic bacteria. Undigested peptides in the gut are broken down by bacteria with proteolytic properties such as *Clostridium, Bacteroides, Fusobacterium, Propionibacterium, Lactobacillus,* and *Streptococcus* (19). Although products such as acetate, butyrate, and propionate (i.e., volatile fatty acids) are part of proteolytic fermentation, the production level is much lower compared to carbohydrate fermentation (20). Cats fed a lower protein, higher carbohydrate diet were found to have higher fecal concentrations of carbohydrate-active enzymes, which are involved in the volatile fatty acid pathways and therefore carbohydrate fermentation (18). The fermentation process to produce volatile fatty acids can also take longer, which may further explain why in the present study the shifts in volatile fatty acid concentrations occurred later (17). In a previous investigation from our lab, Tal et al. (10) did not observe changes in the feline fecal microbial composition across 4 days of room temperature exposure, while the present study (using the same fecal samples) observed changes in feline fecal metabolites 6–24 h post-collection. Therefore, it seems that microbial composition and structure are maintained despite changes in metabolic pathway activity as a result of increased exposure to oxygen at room temperature. Future research can assess the feline fecal microbiota and metabolome simultaneously to verify this point.

The lack of standardized fecal collection, storage, and analysis methods makes comparisons within existing literature difficult. The present study’s findings suggest that exposure of feline fecal samples to room temperature should be limited to no more than 4 h, because beyond that the metabolome composition began to change. In further developing the use of fecal metabolomics as novel diagnostic techniques, sample exposure to room temperatures must be considered when collection methods are out of laboratory settings, such as fecal collection in the field or in a home-setting. Storage conditions post-collection must also be considered. As a fecal sample continues to be exposed to room temperature, the bacteria can continue to consume and produce metabolites, allowing the potential to continually alter the metabolomic profile. Altogether, the time of exposure of fecal samples to above-freezing temperatures prior to storage at −80°C is an important factor to consider in future studies. In a study on humans, fecal samples stored at 4°C demonstrated slowing down of the effects of oxygen exposure, delaying metabolite shifts to 24 h post-collection (8). An important future direction from the present study’s research is to investigate to what extent storage at 4°C can slow metabolite shifts in feline feces as well.

The present study had certain limitations that could be addressed when developing future protocols. The samples were unable to be frozen immediately after defecation, and required extra time for transportation from the boarding facility and for aliquoting, and were therefore exposed to room temperature for an additional hour (hence the use of a 1 h time point but no time point immediately post-collection). Potential metabolite changes during this time could not be detected. The samples in this study were also manually homogenized, which introduces oxygen into the sample that may have enhanced the effect of aerobic fermentation on the fecal metabolome. Future research should explore the effect of room temperature exposure on non-homogenized fecal samples, or in samples stored in an anaerobic environment. Freezing of samples before analysis may also impact the analyses, as changes can occur during the thawing process. Gratton et al. (8) investigated the influence of the freeze–thaw process on human fecal metabolites and found that there were decreased levels of short chained fatty acids and increased levels of branched chain amino acids. They concluded that when transporting samples, it is ideal to minimize the time to prevent thawing, and ideally ship on ice when possible. It is also important to note that variability in the diets of the felines included in the present study was not taken into account. The influence of room temperature exposure on the feline fecal metabolome between diets with different nutrient composition may therefore be a target for future research to further standardize sample collection and storage methods. Finally, the method used for metabolite analysis, ^1^H NMR spectroscopy, was an accurate method to quantify the metabolites of interest, allowing comparisons between several timepoints. However, this is a targeted methodology for detecting known metabolites, and changes in metabolites that have yet to be profiled were undetected (21). Other methodologies for metabolomics analysis such as mass spectroscopy may allow for a wider investigation of metabolites (11).

## 5. Conclusion

This study demonstrated that storage of feline fecal samples at room temperature for the purpose of metabolic profiling using NMR spectrometry can be considered appropriate when these samples are stored for 4 h or less post-collection. Longer exposure to room temperature during collection and storage of fecal samples should be avoided to minimize effects on metabolite concentrations. Future research can compare the influence of room temperature exposure on the feline fecal metabolome between diets with different nutrient composition, and investigate the influence of storage in the fridge post-collection to further standardize sample collection procedures.

## Data availability statement

The original contributions presented in the study are included in the article/supplementary material, further inquiries can be directed to the corresponding author/s.

## Ethics statement

Ethical review and approval was not required for the animal study because the Animal Care Committee at the University of Guelph does not require an Animal Use Protocol for review and approval in cases with no live animal procedures described as part of the conduct of the research. In this case, the procedure of feline fecal collection from the cat litter box, without changing the cats’ normal routine, was not subject for review by the Animal Care Committee. Written informed consent for participation was not obtained from the owners because written informed consent was obtain from the owner of the boarding facility. The researchers did not have contact with the cat owners at any time during the study. Samples were collected by the facility staff and provided to the university research team.

## Author contributions

MT, AV, JW, and MH: conceptualization. MT, AV, AS, and JW: methodology. OC, MH, and DG: validation. MT and AS: formal analysis. OC, MT, AS, and AV: investigation. MT and AV: data curation. OC, MT, and AS: writing – original draft preparation and visualization. MT, JW, DG, MH, and AV: writing – review and editing. AV, JW, and DG: supervision. AV: project administration and funding acquisition. All authors contributed to the article and approved the submitted version.

## Funding

This research as supported by the Natural Sciences Engineering Research Council of Canada, discovery grant (RG PIN-2014-04518).

## Conflict of interest

AV is the Royal Canin Veterinary Diet Endowed Chair in Canine and Feline Clinical Nutrition at the Ontario Veterinary College. MT is currently employed by Royal Canin. The study was conducted before this employment as part of MT’s Doctor Veterinary Sciences Degree and OC’s Master of Sciences Degree at the Ontario Veterinary College.

The remaining authors declare that the research was conducted in the absence of any commercial or financial relationships that could be construed as a potential conflict of interest.

## Publisher’s note

All claims expressed in this article are solely those of the authors and do not necessarily represent those of their affiliated organizations, or those of the publisher, the editors and the reviewers. Any product that may be evaluated in this article, or claim that may be made by its manufacturer, is not guaranteed or endorsed by the publisher.

## Footnotes

^1^Class II, Type A2 Biosafety Cabinet, Thermo Fischer Scientific, Waltham, MA, United States.

^2^University of Alberta Metabolomic Research Services Group, University of Alberta, Edmonton, AB, Canada.

^3^MetaboAnalyst 5.0, 2021, www.metaboanalyst.ca

^4^PubChem, 2021, www.pubchem.nlm.nih.gov
